# The effects of hypnotherapy compared to cognitive behavioral therapy in depression: a NIRS-study using an emotional gait paradigm

**DOI:** 10.1007/s00406-021-01348-7

**Published:** 2022-02-03

**Authors:** Alina Haipt, David Rosenbaum, Kristina Fuhr, Martin Giese, Anil Batra, Ann-Christine Ehlis

**Affiliations:** 1 Tübingen Center for Mental Health (TüCMH), Tuebingen, Germany; 2 Department of Psychiatry and Psychotherapy, University Hospital of Tuebingen, Tuebingen, Germany; 3 Section for Computational Sensomotorics, Department of Cognitive Neurology, Hertie Institute for Clinical Brain Research Centre for Integrative Neuroscience, University Hospital of Tuebingen, Tuebingen, Germany

**Keywords:** Hypnotherapy, Cognitive behavioral therapy, Emotional processing, Temporal lobe, Depression

## Abstract

Hypnotherapy (HT) is a promising approach to treating depression, but so far, no data are available on the neuronal mechanisms of functional reorganization after HT for depressed patients. Here, 75 patients with mild to moderate depression, who received either HT or Cognitive Behavioral Therapy (CBT), were measured before and after therapy using functional near-infrared spectroscopy. We investigated the patients’ cerebral activation during an emotional human gait paradigm. Further, rumination was included as predictor. Our results showed a decrease of functional connectivity (FC) between two regions that are crucial to emotional processing, the Extrastriate Body Area (EBA) and the Superior Temporal Sulcus (STS). This FC decrease was traced back to an activation change throughout therapy in the right STS, not the EBA and was only found in the HT group, depending on rumination: less ruminating HT patients showed a decrease in right STS activation, while highly ruminating patients showed an increase. We carefully propose that this activation change is due to the promotion of emotional experiences during HT, while in CBT a focus lay on activating behavior and changing negative cognitions. HT seemed to have had differential effects on the patients, depending on their rumination style: The increase of right STS activation in highly ruminating patients might mirror the improvement of impaired emotional processing, whilst the decrease of activation in low ruminating patients might reflect a dismissal of an over-compensation, associated with a hyperactivity before therapy. We conclude that HT affects emotional processing and this effect is moderated by rumination.

**Supplementary Information:**

The online version contains supplementary material available at 10.1007/s00406-021-01348-7.

## Introduction

Depression is a widespread mental disorder currently affecting an estimated number of 264 million people worldwide [1]. Besides somatic and emotional symptoms [2] depression is associated with self-referential thoughts of negative content about the past, future and self, called rumination [3, 4]. Cognitive Behavioral Therapy (CBT) has been shown to be an effective treatment for depressed patients [5]. It seems to help normalize depression-specific aberrant prefrontal [6] and amygdala activity [7]. In a meta-analysis on the neurophysiological effects of depression treatment, the authors report effects in frontal areas bilaterally and the lingual gyrus, middle temporal gyrus and middle cingulate cortex left-hemispherically [8]. The effectiveness of CBT in treating depression might originate in an increase of prefrontal functioning, which is associated with cognitive control [9, 10]. On the other hand, Hypnotherapy (HT), as one of the oldest techniques used in treating mental and somatic disorders [11], displays a promising alternative to CBT in reducing depressive symptoms [12]. Meta-analyses have substantiated the adjuvant effect of hypnotic interventions in treating depressed patients [13, 14] and greater effect sizes in symptom reduction when HT is added to CBT compared to sole CBT elements [15]. Fuhr et al. [16] showed in their WIKI-D study that HT is similarly effective to CBT in reducing mild to moderate depression despite their obvious theoretical and conceptual differences. The neurophysiological effects of HT were investigated in patients with irritable bowel syndrome [17] and dental phobia [18]; in recent literature, the neurophysiological effects of hypnosis and HT are reviewed and a biopsychosocial model of hypnosis was suggested [19, 20]. However, so far, the underlying neurobiological processes of HT applied specifically for treating depressed patients have not been investigated.

Depressed patients show altered emotional processing on a behavioral and perceptional level, including aberrant emotional recognition [21, 22], a negativity bias [23, 24], as well as attention biases [25, 26]. Strikingly, most studies on emotional recognition in general and affective perception during depression have been conducted with faces as stimulus material [27]. However, humans are often forced to use other clues than faces, such as posture or gait [28], to infer emotional states in others. Thus, in this study, we focus on the ability of emotional recognition based on human gait.

Neuroanatomically, emotion recognition based on whole-body movements is related to the Extrastriate Body Area (EBA) in the posterior inferior temporal sulcus/middle temporal gyrus [29–32]. Another region of interest (ROI) is the superior temporal sulcus (STS), which is associated with the analysis of biological motion [33–39] and also theory of mind (ToM) processes [39] like understanding the actions [40–43] or mental states of others [44–48]. It was found to be active when presenting subjects with dynamic emotional body expressions [28, 49–51]. Moreover, depression is associated with abnormalities in the superior temporal cortex (including the superior temporal gyrus (STG) and its lateral part, which is often referred to as STS region [52]). In a meta-analysis of functional magnetic resonance imaging (fMRI) studies, the authors found the STG to be one of the regions most consistently involved in depression [53–56].

In the past years, research has focused on investigating connections between cerebral areas (i.e., neural networks) rather than isolated brain regions. Consequently, in this study, we concentrated on the functional co-activation of distinct brain areas—namely the STS and EBA—in terms of functional connectivity (FC). From a theoretical perspective, emotion recognition based on human gait should be reflected in an increased FC between STS and EBA, since both areas are associated with motion recognition as well as emotional processing (STS) and dynamic emotion recognition (EBA), as described above. A coupling of these two areas could reflect a process of the sensory perception of a dynamic emotional body expression (EBA activation) as well as its integration and interpretation (STS activation).

Based on previous own research including a subsample of patients used in this study, it seems that the extent to which depressed patients ruminate influences cerebral activation [57]. Depressed patients who react habitually with rumination to sad mood (trait rumination) showed less FC within the Default-Mode Network (DMN), a major cerebral network that is associated with processes relevant to depression [58–60].

Functional near-infrared spectroscopy (NIRS) is a non-invasive method for optically based functional imaging, offering many advantages including its easy and quick applicability in a noise-free setting and its (relative) tolerance towards movement [61, 62], as well as few exclusion criteria. NIRS has been shown to be an apt method to measure activation changes in cerebral networks [57, 58].

Based on the theoretical and empirical background outlined above, we focused on the effects of CBT vs. HT on neurophysiological correlates of emotional processing. We also explored potential moderating influences of rumination and additionally analyzed emotion recognition as a behavioral correlate of emotional processing. Therefore, we examined a subsample of depressed patients who participated in the WIKI-D study [16] before and after therapy (CBT or HT). Using a human gait paradigm [28], activation in cortical areas associated with emotional processing and depression (EBA, STS) was elicited and measured with NIRS. Due to the exploratory nature of this study, our main hypothesis was that changes in the STS-EBA network associated with emotional processing would occur over the study period and that these changes would differ between CBT and HT treatment. Due to previous own research, we further hypothesized that rumination would moderate this change in connectivity between and among the groups and that the cerebral effects would be mirrored in the patients’ behavior.

## Methods

### Subjects

**Fig. 1:**
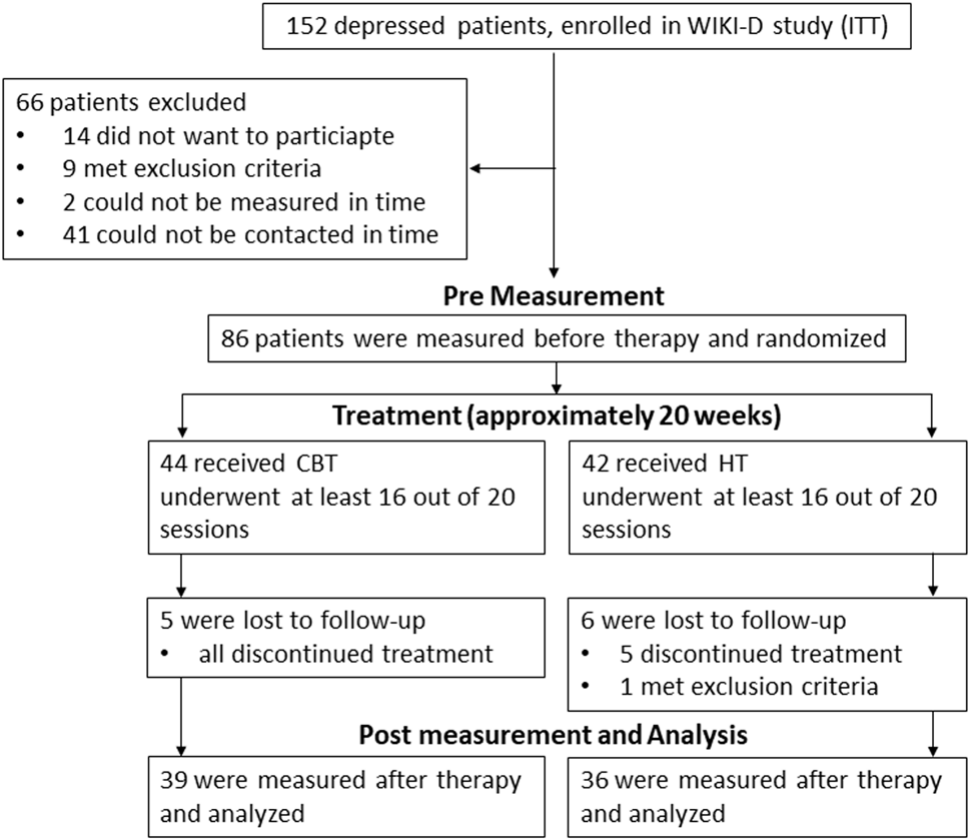
Procedure of including and measuring patients throughout our study. Number of patients in the WIKI-D study [16] (N); intended to treat (ITT); Cognitive Behavioral Therapy (CBT); Hypnotherapy (HT)

All participants were recruited from 152 patients (intended to treat) of the WIKI-D study [16], all being diagnosed with a unipolar mild to moderate depressive episode by trained clinicians using the Structured Clinical Interview for DSM-IV (SKID-I; [63]). Exclusion criteria for our neurophysiological measurements were pregnancy or nursing a child, severe neurological diseases (e.g., meningitis, epilepsy), untreated hypertension, diabetes, or other coronary diseases as well as social phobia. In total, we included 75 patients (56 female, 19 male; 18–69 years, *M* = 39.24, *SD* = 14.85) participating in 2 NIRS measurements, 1 before and 1 after therapy (Fig. 1). Nearly one third of the patient sample (*n* = 24) showed at least 1 acute comorbid disorder; 25 patients (*n* = 25) took at least 1 antidepressant medication (36% SSRIs or other including atypical antipsychotics^1^), which had to have been taken without changes for 3 months prior to the study. All participants gave their written informed consent to participate in the study.

### Stimuli and materials

To test affective processing, we used an emotional gait paradigm [28, 64], portraying sad, happy and neutral stimuli. We measured NIRS data as well as the reaction time and errors on a behavioral level. The happy gait was faster than the sad gait; the neutral walk was presented in three velocities (fast, medium, slow). The displayed video clips (3 s each) showed dark grey volumetric avatars walking across the screen from left to right or vice versa (at an angle of 22°). The avatars were uninformative about age, race or sex and still appeared human. The emotional videos (sad and happy) were presented 12 times each, the neutral videos (fast, medium, slow) 4 times each, once for each direction. This resulted in 72 videos total, intermitted by a break. Within blocks, the presentation of the videos was randomized. Rumination as a trait variable was assessed with the Rumination Response Scale [65].

### Procedure

Measurements took place after diagnostics and before undergoing psychotherapy, and after therapy completion (Fig. 1). Patients were randomly assigned to either CBT (*n* = 39) or HT (*n* = 36). The therapy was considered completed when patients underwent at least 16 of the 20 sessions, which applied to 76 patients. One patient became pregnant during therapy and was excluded from the second measurement. Each NIRS measurement lasted 2 h and included two additional paradigms besides the gait paradigm, the results of which will be reported elsewhere. Moreover, RRS data were collected between paradigm presentations.

During the measurement, oxygenated (O_2_Hb) and deoxygenated hemoglobin (HHb) were recorded continuously after a 10 s baseline measurement. Subjects were asked to judge the portrayed emotion of a walking avatar by pressing the allocated response button (arrow buttons: left, down, and right). The down button always corresponded to neutral gait, the left and right buttons served as response buttons for sad or happy gait (balanced across subjects). Responses could be given as soon as the emotion was recognized. Subjects were not specifically asked to answer as quickly as possible to promote emotional processing over guessing. After a practice trial with feedback, the main experiment began. Each trial started with a fixation cross in the middle of the computer screen (400 ms), followed by a blank screen (100 ms) and an avatar video clip (2000 ms), which was followed by 200 ms blank screen. The duration of the inter-trial interval varied randomly among 5000 and 9000 ms. After 36 trials, the subjects were given a break, which they could end themselves. The experiment lasted about 18–20 min. All subjects received a small monetary compensation for their time.

### Near-infrared spectroscopy and regions of interest

Light in the near-infrared spectrum can penetrate the skull and other biological tissue. Depending on the O_2_Hb and HHb in the underlying brain tissue, the light is absorbed differently and thus indicates the relative concentration of both chromophores. From this concentration conclusions can be drawn on cortical activation levels underneath the measurement probes. In our study we used an ETG-4000 Optical Topography System (Hitachi Medical Corporation, Tokyo, Japan) with a 52-channel array of 33 optodes (17 light emitters and 16 detectors) covering posterior-occipital and temporal brain areas (temporal resolution: 10 Hz). The inter-optode distance was 3 cm and near-infrared light with two wavelengths (695 and 830 nm) was used. The optodes were arranged in a 3 × 11-rectangular shape on the subjects’ heads in respect to the international 10/20 System [66]. Channel 37 (middle channel in the lowest channel row) was placed over Oz; the anterior channels 43 (left) and 52 (right) were positioned towards the temporal positions T3 and T4, respectively. To assign the channel positions to the anatomy, a neuronavigation system was used on a volunteer’s head.

**Fig. 2:**
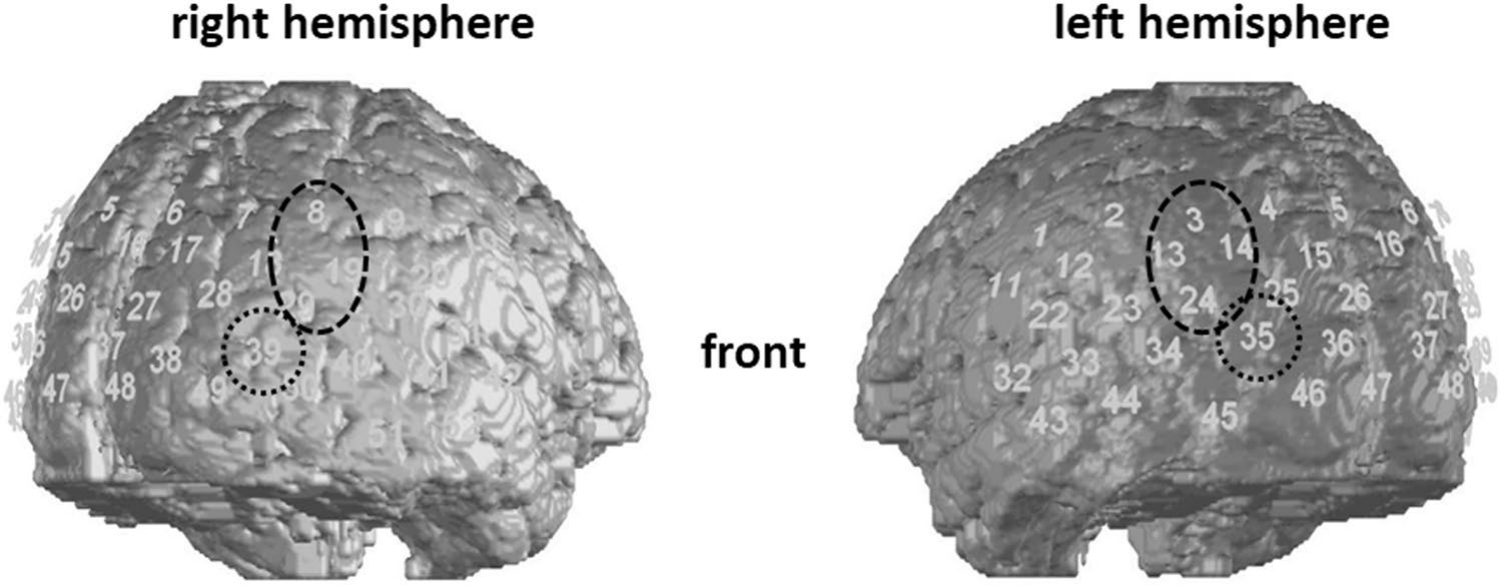
Regions of Interest in the right and left cerebral hemisphere. The dashed circles portray the STS region (Channels: 8, 19, 29 (right); 3, 13, 14, 24 (left)), the dotted circles the EBA region (Channels: 39 (right); 35 (left)). Superior Temporal Sulcus (STS); Extrastriate Body Area (EBA)

We selected ROIs according to previous findings [28]: namely the EBA and the STS region (including the STG; Fig. 2). Due to limited spatial resolution in NIRS [67, 68], we do not distinguish between activation due to motion perception or emotional perception—both evoking activation in the STS.

### Data analyses

#### Preprocessing

The recorded NIRS data were preprocessed and brain plots were generated using MATLAB R2017b (MathWorks Inc, Natick, USA). Preprocessing included applying an algorithm for movement artefact reduction [69]. Then, all signals were visually inspected for local artefacts, 3% of the channels in the pre data and 4% in the post data were interpolated from adjacent channels. All signals were then scanned for biting artefacts and contaminated events were excluded from further analysis. We applied a band-pass filter (0.008–0.25 Hz) to minimize high- and low-frequency noise. Signals were z-transformed to compare between subjects, then the FC between STS and EBA was calculated. To explain the FC effects, post hoc analyses were conducted on the ROIs separately. For this, the NIRS signals were fitted to a model hemodynamic response function in a general linear model based regression [28, 70]. Based on previous studies using the same paradigm (e.g., [28]) as well as visual inspection of the current data set, peak time was set to 12 s with a peak dispersion of 2 s. In the behavioral data, responses given in under 300 ms were excluded because we do not expect them to result from cognitive processing.

#### Statistics

For the behavioral and NIRS data analyses, as well as non-brain figures, we used R Studio (R Studio Inc, Boston, USA). To account for the STS-EBA FC pre therapy and the therapy effect concurrently, we calculated the change score (CS): FC(post therapy)—FC (pre therapy), which served as dependent variable. Linear regression models were constructed consecutively adding fixed effects (first additively, then multiplicatively), in this order: FC(pre), therapy group (CBT = 0 vs. HT = 1), rumination (continuous variable) and portrayed emotion (neutral, sad, happy). The models were compared using F-tests. The most complex model accounting for significantly more variance than the previous model was selected. The regression models were constructed in the following way:

$${\text{CS}}\, = \,\beta _{0} \, + \,\beta _{i} \times {\text{ }}X\, + \,\varepsilon .$$

We applied the same regression analyses, including the significant predictors, on the behavioral data to capture changes in emotion recognition. We calculated the relative error rate (RER) (sum of errors/trial number) and its CS (RER post therapy–RER pre therapy), which served as dependent variable in regression analyses. Reaction times were looked at on a descriptive level, since they could not be interpreted: no instruction was given to answer as quickly as possible. Post hoc two-tailed t-tests or correlation tests were conducted to further analyze interaction effects. Level of significance was *α* = 0.05.

## Results

In the left hemisphere, the linear model containing pre FC as an additive term was the best fitting model for the CS, explaining a significant proportion of variance (*F*(1,223) = 169.20, *p* < 0.001, *r*^*2*^ = 0.43). No further predictors contributed significantly. The FC before therapy significantly predicted the FC change in the left hemisphere (*ß* = − 0.78, *t*(223) =  − 13.01, *p* < 0.001): The lower the FC between STS and EBA before therapy, the more it increased and vice versa (Online Resource 1). This effect could reflect a regression towards the mean effect, but also regulatory changes over time: patients who showed little EBA–STS coupling at baseline developed this coupling over therapy; patients who had lots of coupling between these two ROIs, possibly reflecting an overcompensation, showed decreased FC.

**Fig. 3:**
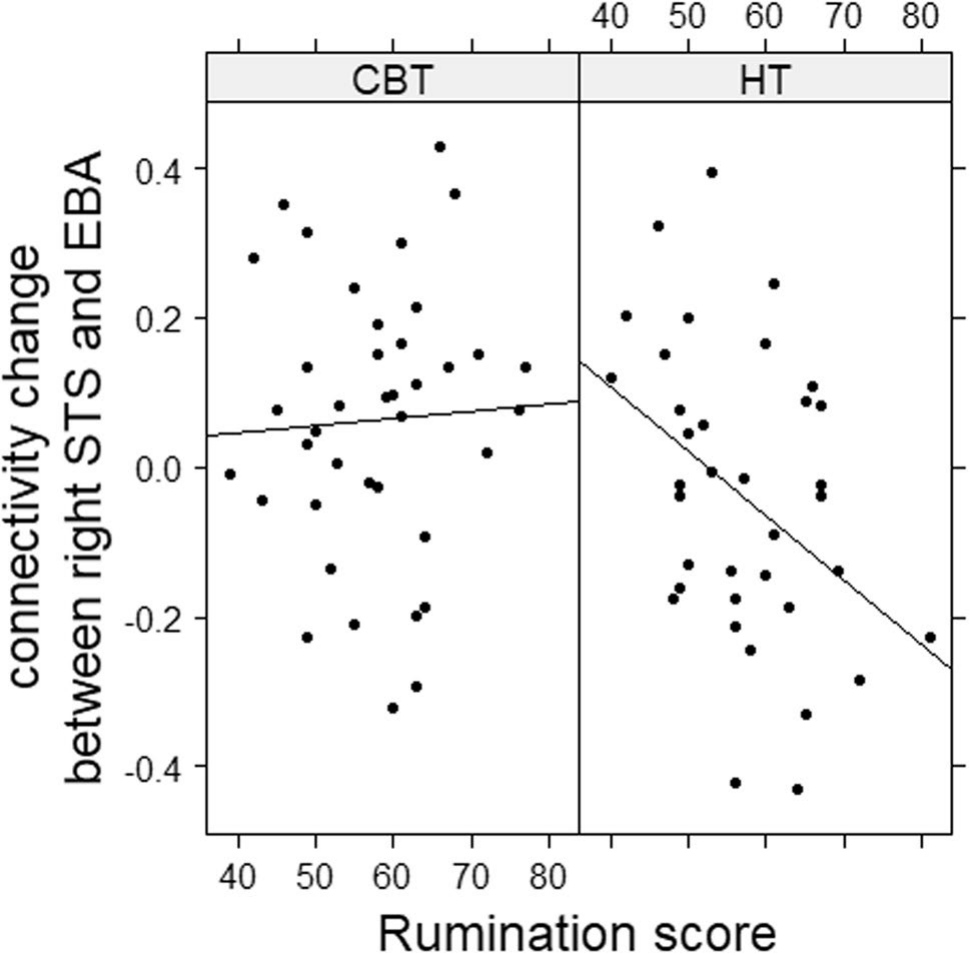
Correlation between the change of connectivity between the right STS and EBA separately for the two therapy groups. Superior Temporal Sulcus (STS); Extrastriate Body Area (EBA); Cognitive Behavioral Therapy (CBT); Hypnotherapy (HT)

For the right hemisphere, the model on the CS including pre FC as additive term and rumination and group as multiplicative predictors was the most complex model contributing significantly to the explained variance (*F*(4, 220) = 42.30, *p* < 0.001, *r*^*2*^ = 0.42). This model revealed a significant influence of pre FC (*ß* = − 0.70, *t*(220) =  − 11.21, *p* < 0.001), group (*ß* = 0.37, *t*(220) = 2.60, *p* = 0.01) and an interaction between rumination and group (*ß* = − 0.01, *t*(220) =  − 3.04, *p* = 0.003). The pre-FC effect implies, again, a negative connection between pre FC and the CS, thus portraying a regression to the mean on an analytical level and a possible compensation effect on a content level. The main effect for therapy group reflects a change in FC throughout therapy only in the HT group, while the interaction effect further specifies this change within the HT group, depending on rumination. To better understand this interaction effect, we looked at the association between rumination and the CS separately for both groups. In the CBT group, there was a non-significant positive correlation between rumination and the CS (*r* (37) = 0.05, *p* = 0.77), while there was a significant negative correlation (*r*(34) = − 0.40, *p* = 0.02) in the HT group (Fig. 3). Since the predictor “emotion”, accounting for the emotional valence of the portrayed gait, did not add significantly to the explanation of variance, the data were pooled across all conditions (happy, sad, and neutral) for further analyses.

**Fig. 4:**
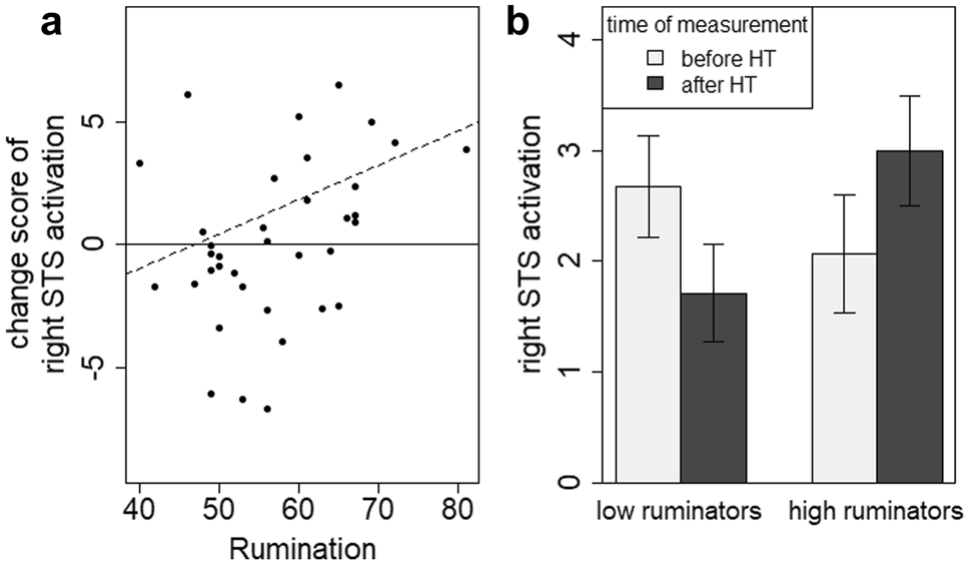
**a** CS below and above zero (black line) and the regression line (dashed line) between the CS of right STS activation and rumination, the result of our regression analysis (including preactivation as additive predictor). **b** Categorial illustration of the association between rumination and the activation of the right STS in the HT group. Change Score (CS; Superior Temporal Sulcus (STS); Hypnotherapy (HT)

**Fig. 5:**
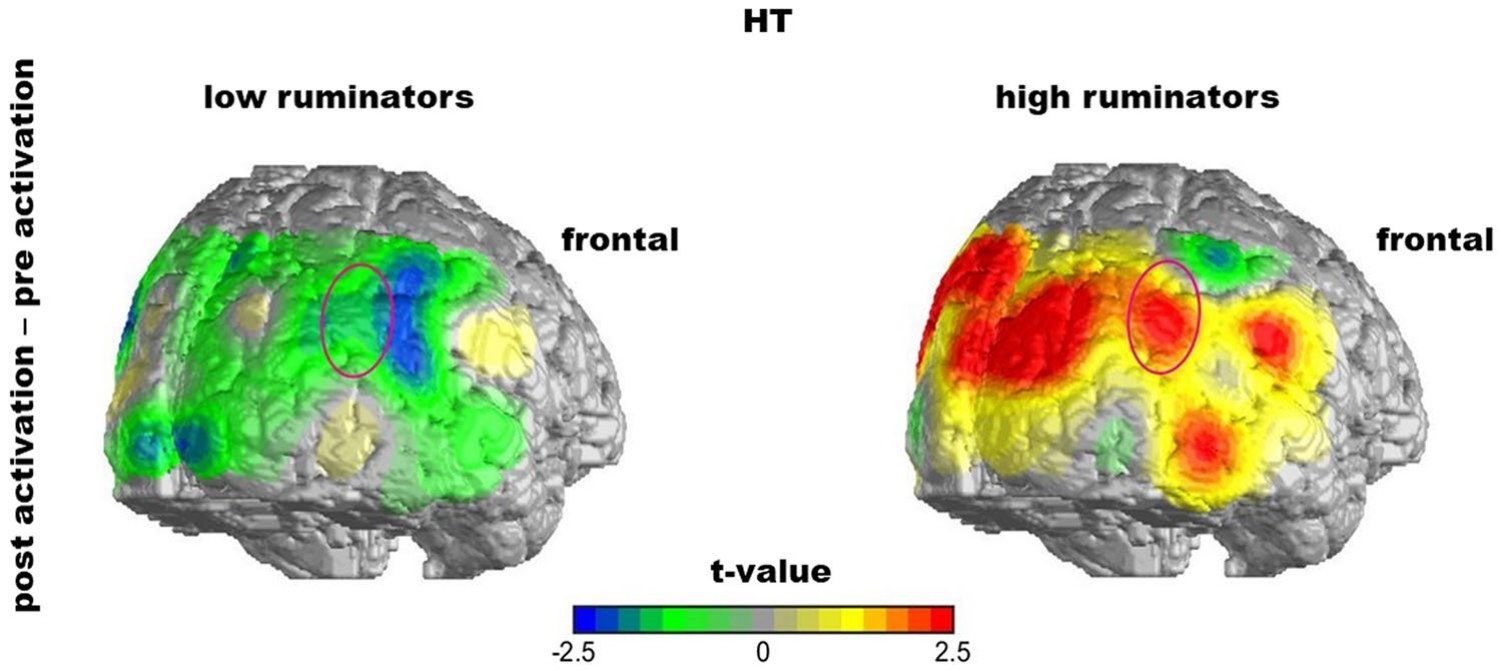
The effect of rumination on the change of STS activation throughout therapy in the HT group. The figure portrays a t-test between activation before therapy (pre) compared to after therapy (post) (post–pre) in the HT group for either low or high ruminators. Superior Temporal Sulcus (STS); Hypnotherapy (HT)

To further uncover neurophysiological changes underlying the right hemispheric decrease of FC in the HT group, we conducted post hoc analyses on the activation of the right STS and EBA separately in the HT group. For both ROIs we followed the same regression approach constructing the models consecutively, CS being the dependent variable and adding preactivation and rumination as fixed effects. As assumed, for the right STS the model including preactivation and rumination yielded significance (*F*(2,33) = 13.84, *p* < 0.001, *r*^*2*^ = 0.42) and revealed main effects for preactivation (*ß* = − 0.64, *t*(33) = -4.50, *p* < 0.001) as well as rumination (*ß* = 0.14, *t*(33) = 3.01, *p* = 0.005) on the CS (which results in a significant correlation between rumination and CS of *r*(34) = 0.35, *p* = 0.04) indicating an increase in activation for those patients that showed less activation before HT and vice versa, equivalent to the previous analyses. This, again, could show a regression to the mean and possibly a compensation effect. The rumination effect indicates that higher levels of rumination were associated with a greater increase in right STS activation when patients received HT (Fig. 4a). Considering the distribution of CS, it became apparent that negative changes (i.e., a decrease) in right STS activation occurred in a rather large subgroup of patients (who tended to show lower trait rumination scores; Fig. 4a), while positive changes appeared more often in patients that tended to ruminate more. This might suggest a categorical relation between rumination and activation changes rather than a continuous one. For illustration purposes, this categorial relation is portrayed in Figs. 4b and 5.

For the activation in the right EBA in the HT group, no model including any predictors contributed to the explanation of variance implying no systematic change of activation in the right EBA in the HT group. We conclude that the decrease in right STS–EBA connectivity in the HT group could be derived from a change in activation in the right STS. Further, the STS activation change depended on rumination: while patients with rather low rumination scores showed decreased right STS activation after therapy, this effect was reverse for “high ruminators”. Therefore, we explain the decrease in right STS–EBA connectivity with this differential STS activation effect. Next, and for the purpose of completion, we checked for the activation in the right STS before therapy and whether it differentiated between the groups to rule out possible group effects due to different preactivation. We did not find a difference in right STS activation between groups before therapy (*M*(HT) = 2.24, *SD*(HT) = 3.02; *M*(CBT) = 2.53, *SD*(CBT) = 3.00; *t*(73) = 0.41, *p* = 0.69). Finally, as an additional post hoc analysis, we correlated rumination with self-reported symptoms before therapy in both groups and found a significant correlation (*r*(73) = 0.33, *p* = 0.004). This offers an alternative explanation for the HT effect: The effect of HT could originate in initial symptom severity which is linked to rumination.

### Behavioral data

On a descriptive level, reaction times decreased from before to after therapy, and in the HT group there is a slight positive correlation between reaction time and rumination (Online Resource 2). For the RER, descriptive parameters show a decrease over time (as can be seen in the tables, Online Resource 3). Further analysis revealed no systematic connection between RER, rumination, and groups. Applying the same regression method (stepwise regression, excluding the factor “emotion”), the model explaining a significant amount of variance (*F*(1,65) = 92.75, *p* < 0.001, *r*^*2*^ = 0.58) included the RER before therapy to be the only predictor of the CS of RER (*ß* = − 0.90, *t*(65) =  − 9.63, *p* < 0.001). This result was consistent, even when 2 outliers were excluded from the analysis (*F*(1, 63) = 12.82, *p* = 0.001, *r*^*2*^ = 0.12, RER before therapy as predictor: *ß* = − 0.52, *t*(63) =  − 3.58 *p* = 0.001). Thus, patients who either made very few or many mistakes at baseline showed a bigger change in their error rate after therapy (resulting in fewer mistakes for those who had many mistakes at first or more mistakes for those who had few mistakes before therapy). This seems to reflect a typical regression to the mean effect and partly a floor effect since the RER could not sink below zero. Since we did not find group- or rumination-specific effects in the behavioral data, we concluded that the activation change in the right STS in the HT group did not reflect the patients’ behavior.

## Discussion

This study addressed the question whether two psychotherapies (CBT and HT) used in depression treatment affect the brain differently. Since the content of CBT and HT differs widely, particularly regarding the work with emotions, we hypothesized that these two therapies differ in their effect on a cerebral network—consisting of the STS and EBA—that is associated with emotional processing. Our main results show a decrease in right hemispheric STS–EBA connectivity in patients who received HT. This decrease in FC can mainly be explained by a change of activation in the STS while the EBA did not change in its activation. This activation change in the right STS in the HT group depended on rumination: while subjects with lower rumination scores showed a decrease in their right STS activation, subjects with higher rumination scores showed an activation increase. These specific neurophysiological changes were not mirrored in behavioral data and were independent from the type of displayed emotion.

These results have multiple implications. First, even though both the STS and EBA were previously found to be active during displayed biological motion [28, 31, 33, 34, 39, 49, 71, 72], here, only the right STS showed activation changes throughout therapy. However, since we focus on the co-activation of the EBA and STS, we cannot make firm conclusions about the activation of the EBA during the gait paradigm by itself. Based on our analysis, EBA activation did not change throughout therapy, suggesting it might not be involved in emotional processes changing in psychotherapy; the STS activation change, however, might be related to these emotional processes. The STS region has shown to be involved in pathological brain activation in depressed patients, when confronted with negative emotional stimuli, with clear lateralization, though [53]. The findings of STS activation in response to perceived body motion were heterogenous in regard to lateralization [28, 33, 34, 39, 72]. Thus, the STS seems to play a role in emotional processing influenced by depression-specific HT; the role of lateralization remains to be investigated.

Interestingly, this STS activation change throughout therapy was only found in the HT group. Since the groups did not differ in right STS activation before therapy, the group effect of the FC change is due to the differential activation after therapy. This difference could be due to the increased focus on emotional therapeutic work in HT compared to CBT. In CBT, the focus lay on activating, analyzing and reviewing behavior and recognizing and changing negative cognitions [73]. This was implemented during therapy sessions and homework. In CBT emotions were mostly addressed on a conversational level. On the other hand, in HT, a focus lay on using the patients’ own strengths, positive experiences and memories to create alternatives to negative, wearing thoughts. Namely, pleasant emotions linked to positive personal experiences were induced during trance to make the patients feel competent, hopeful, and strong. The trances were recorded and handed to the patients to listen to them again at home. This might have led to a repetition of the emotional experiences. We argue that HT promoted changing one’s perspective since multiple trances focused on putting oneself in a former or later self, possibly reflecting ToM processes. Since the STS region is associated with these emotional and ToM processes [39, 53] it seems fitting that right STS activation changed throughout HT, but not CBT. CBT, on the other hand, has been shown to normalize aberrant amygdala [7] and prefrontal [6] activity, the latter being associated with cognitive control [9, 10, 74].

Interestingly, hypnosis has been shown to lead to decreased prefrontal activity [89, 90] and disrupted prefrontal FC [91]. Therefore, the PFC seems to be important to consider when searching for the mechanisms underlying therapeutic effects of HT. Our research group investigated prefrontal areas in the same study sample as presented here (using a different paradigm and NIRS cap placement); the results will be published elsewhere.

Secondly, we found that the right STS activation change after HT depended on the patients’ rumination: Patients who tended to ruminate less before therapy showed a decrease while patients who tended to ruminate more showed an increase in right STS activation after HT suggesting a categorical division of patients in high and low ruminators. This is in line with previous research of our group, in which differential DMN activity was found for high versus low ruminating depressed patients [57]. In previous research, trait rumination was shown to be positively associated with depressive symptoms and their severity [76–77], mediating risk factors for depression [78], and predicting treatment success [79]. We, as well, found a correlation between rumination and self-reported symptoms. Further research has shown abnormal functioning of the DMN in depressed subjects [60] and decreased FC within the DMN in depressed subjects who tended to ruminate [57]. The DMN also seems to be linked to ToM and thus emotional processes, which elicit increased activity in lateral regions like the STS and increased coupling with the medial prefrontal cortex, a core node of the DMN [80]. Furthermore, multiple authors suggested a link between hypnotic trance and the DMN e.g., [77–80]. More detailed results on the connection between HT and the DMN will be derived from a resting-state measurement also conducted in the context of this study and published elsewhere. In line with this research, our results might indicate a greater degree of depression in highly ruminating patients whose decreased STS activity before therapy possibly reflected impaired emotional processing. The increase of STS activity throughout HT in these patients could be a neurophysiological correlate of normalized emotional processing induced by HT as described above. On the other hand, low ruminating depressed patients showed a decrease in STS activity throughout HT. Our results propose that low ruminators show less severe depressive symptoms and might therefore be less impaired in emotional processing. Opposed to the more severely impaired patients, the less impaired might still be capable to compensate abnormal emotional processing with a certain effort. This compensation effort could be associated with hyperactivity in the right STS. The benefit of HT for these patients could be linked to a decrease in right STS activity due to the reduction of over-compensation. Further research is needed to underpin this hypothesis and clarify the interaction of rumination and symptom severity on depression-related cerebral activity.

Thirdly, the absence of significant predictive value of the factors group and rumination for the error rate (RER) implies that activity changes in the right STS did not influence the behavioral data in a measurable way. A possible explanation is that the RER is based on the reaction as a consequence of emotional recognition. This reaction is rather a judgement than a mirror of the preceding emotional processing. Emotional judgement, though, is associated with prefrontal activity [85–87] and might not be correlated with temporal activity. To get to the core of this effect, further research with more specific hypotheses is needed.

Lastly, the portrayed emotion did not predict the FC change between EBA and STS throughout therapy nor did it predict the activity change in the right STS. In previous studies, differences in EBA activation occurred between negative emotional body expressions compared to neutral expressions [28, 49, 71, 72], but all of these studies were conducted with healthy subjects. Indeed, depressed subjects have shown aberrant emotional processing on a behavioral level compared to healthy controls [22–24, 26, 88], but in these studies cerebral activation was not assessed. The cerebral processes measured in our study and the changes throughout HT do not seem to be emotion-specific.

### Study limitations and future research

To our knowledge, no previous study has investigated the FC of these two regions even though they were found to be involved in the processing of dynamic, emotional body expressions, and none of them including depressed subjects [28, 49, 71]. This present study is of an exploratory nature and consequently more research is needed to investigate the FC between STS and EBA in depressed subjects. Moreover, other regions involved in the processing of emotional dynamic body expression, like the amygdala [49], cannot be measured with NIRS. Furthermore, due its relatively low spatial resolution, NIRS might not be an adequate method to distinguish motion perception and emotional perception, which are both associated with activation in the STG. e.g., [35, 36]. Therefore, interpretations about emotional processing associated with STS activity need to be considered carefully. To further investigate the co-activation of other areas, including subcortical ones, and to distinguish intra-STS areas, fMRI data should be obtained. Another methodological restriction is that we could not perform a short-channel regression since our NIRS system does not allow to routinely include short-distance channels. Therefore, we cannot exclude the possibility that superficial perfusion changes contributed to our NIRS data. A further limitation is that systemic effects were not controlled during data preprocessing which might have influenced the results.

The assumption that HT fosters more emotional processes than CBT was based on manuals used in the WIKI-D study [16] and interviews with experts. We do not claim this to be the only differentiating factor between HT and CBT; further research is required to clarify the differences. In the future, rumination should be continuously included when investigating depression as it seems to be linked to abnormal, depression-specific, cerebral functioning [57]. This would help to better understand depression in its many facets and to individualize treatment.

### Conclusion

This first of its kind study explores the cerebral processes underlying differential effects of Hypnotherapy (HT) and Cognitive Behavioral Therapy (CBT) in treating depressed patients. In the treatment of depressed patients, HT was not inferior to CBT [16]. Still, how these treatments work (or which group pf patients responds well to either type of therapy) remained unclear. In our study, we investigated the effects of HT and CBT on cerebral activation associated with emotional processing. We found that activation in the right STS changed throughout therapy specifically in the HT group. Further, rumination played a crucial role in predicting activation changes in the HT group and thus seems to be an important factor to consider when drawing conclusions about depression treatment and differential indication. We conclude that HT affects emotional processing in depressed patients and this effect is moderated by the patients’ rumination style. Depression-specific HT, its content, effects and underlying processes have rarely been investigated and therefore many of our assumptions were exploratory in nature. This paper was designed to be a first step in the direction of exploring HT and its differential effects.

## Supplementary Information

Below is the link to the electronic supplementary material.

Supplementary file1 (TIF 675 KB) Online Resource 1. Change of connectivity between the STS and EBA of the left hemisphere depending on the connectivity before therapy. Since “emotion” did not yield significance as predictor, this factor was excluded from this graph. Superior Temporal Sulcus (STS); Extrastriate Body Area (EBA)

Supplementary file2 (TIF 675 KB) Online Resource 2. Reaction times for patients in the different therapy groups CBT and HT depending on rumination. The line portrays the correlation between rumination and reaction time. Data are pooled across “Emotion”. Cognitive Behavioral Therapy (CBT); Hypnotherapy (HT).

Supplementary file3 (DOCX 20 KB)

Supplementary file4 (PDF 456 KB)

Supplementary file5 (PDF 286 KB)

## Data Availability

The datasets from the current study are available upon request.

## Footnotes

1 Besides NaSSA, tricyclic antidepressants, hypericum, SSNRI, agomelatin, bupropion, anticonvulsive medication.

## Code availability

The codes of the current study are available upon request.
