# Supplementary figures and images for: The effects of hypnotherapy compared to cognitive behavioral therapy in depression: a NIRS-study using an emotional gait paradigm
Source: Eur Arch Psychiatry Clin Neurosci. 2022 Feb 3;272(4):729–39. doi: 10.1007/s00406-021-01348-7 (PMC9095550; doi:10.1007/s00406-021-01348-7)

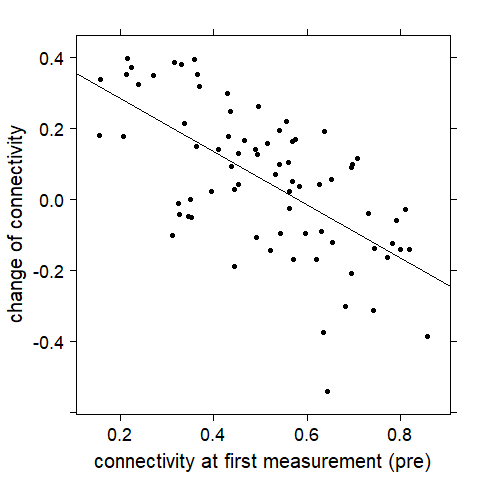

Supplement: Supplementary file 1 — Supplementary file1 (TIF 675 KB) Online Resource 1. Change of connectivity between the STS and EBA of the left hemisphere depending on the connectivity before therapy. Since “emotion” did not yield significance as predictor, this factor was excluded from this graph. Superior Temporal Sulcus (STS); Extrastriate Body Area (EBA) [file 406_2021_1348_MOESM1_ESM.tif]

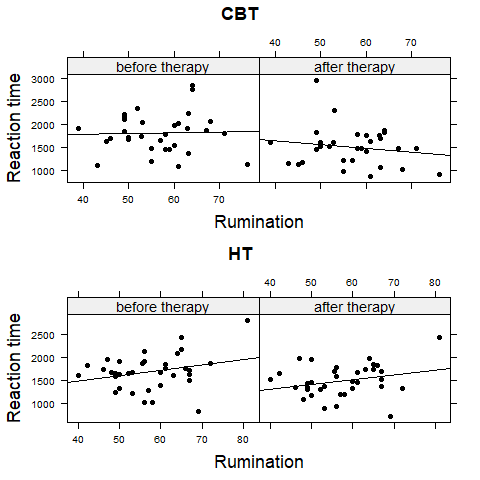

Supplement: Supplementary file 2 — Supplementary file2 (TIF 675 KB) Online Resource 2. Reaction times for patients in the different therapy groups CBT and HT depending on rumination. The line portrays the correlation between rumination and reaction time. Data are pooled across “Emotion”. Cognitive Behavioral Therapy (CBT); Hypnotherapy (HT). [file 406_2021_1348_MOESM2_ESM.tif]
