# Supplementary material for: The effects of hypnotherapy compared to cognitive behavioral therapy in depression: a NIRS-study using an emotional gait paradigm
Source: Eur Arch Psychiatry Clin Neurosci. 2022 Feb 3;272(4):729–39. doi: 10.1007/s00406-021-01348-7 (PMC9095550; doi:10.1007/s00406-021-01348-7)
Supplement: Supplementary file 3 — Supplementary file3 (DOCX 20 KB) [file 406_2021_1348_MOESM3_ESM.docx]

**Supplementary Information**

**Article:** “The Effects of Hypnotherapy on emotion-related processes in the temporal lobe”

**Journal:** “European Archives of Psychiatry and Clinical Neuroscience”

**Authors:** Alina Haipt*, David Rosenbaum, Kristina Fuhr, Martin Giese, Anil Batra, Ann-Christine Ehlis

*Alina Haipt, ORCID: 0000-0003-2506-4556, Department of Psychophysiology and Optical Imaging, University Hospital of Tuebingen

E-mail: alina.haipt@med.uni-tuebingen.de; alinahaipt@gmail.com

**Online Resource 3**

| *Mean* |  |  | | Emotion | |  | | overall | |
| --- | --- | --- | --- | --- | --- | --- | --- | --- | --- |
|  | | happy | | neutral | | sad | |  |  |
|  |  | RT | RER | RT | RER | RT | RER | RT | RER |
| Overall | Before therapy | 1809.01 | 0.31 | 1950.70 | 0.34 | 1460.07 | 0.06 | 1739.93 | 0.24 |
|  | After therapy | 1553.77 | 0.25 | 1706.03 | 0.36 | 1227.86 | 0.04 | 1495.89 | 0.22 |
| HT | Before therapy | 1775.67 | 0.33 | 1938.38 | 0.37 | 1344.38 | 0.04 | 1686.15 | 0.25 |
|  | After therapy | 1545.15 | 0.23 | 1726.89 | 0.38 | 1207.91 | 0.04 | 1493.32 | 0.21 |
| CBT | Before therapy | 1845.47 | 0.30 | 1964.17 | 0.30 | 1586.60 | 0.08 | 1798.75 | 0.23 |
|  | After therapy | 1563.19 | 0.28 | 1683.22 | 0.33 | 1249.69 | 0.04 | 1498.70 | 0.22 |

Online Resource 3. Table 1. Mean values of the behavioral data including the reaction time (RT) and the relative error rate (RER). The values are portrayed for all conditions, both groups, before and after therapy. Cognitive Behavioral Therapy (CBT); Hypnotherapy (HT).

| *SD* | Emotion | | | | | | | overall | |
| --- | --- | --- | --- | --- | --- | --- | --- | --- | --- |
|  | | happy | | neutral | | sad | |  |  |
|  |  | RT | RER | RT | RER | RT | RER | RT | RER |
| Overall | Before therapy | 486.66 | 0.19 | 462.25 | 0.16 | 455.07 | 0.15 | 509.64 | 0.21 |
|  | After therapy | 431.12 | 0.20 | 451.75 | 0.18 | 382.44 | 0.07 | 465.76 | 0.21 |
| HT | Before therapy | 485.06 | 0.17 | 451.58 | 0.17 | 398.69 | 0.09 | 506.56 | 0.21 |
|  | After therapy | 417.61 | 0.21 | 449.00 | 0.19 | 323.71 | 0.06 | 451.51 | 0.21 |
| CBT | Before therapy | 493.52 | 0.21 | 480.53 | 0.15 | 484.70 | 0.19 | 508.89 | 0.21 |
|  | After therapy | 451.96 | 0.18 | 460.82 | 0.17 | 442.18 | 0.07 | 483.23 | 0.20 |

Online Resource 3. Table 2. Standard deviations (SD) of the behavioral data including the reaction time (RT) and the relative error rate (RER). The values are portrayed for all conditions, both groups, before and after therapy. Cognitive Behavioral Therapy (CBT); Hypnotherapy (HT).
