# Supplementary material for: The effects of hypnotherapy compared to cognitive behavioral therapy in depression: a NIRS-study using an emotional gait paradigm
Source: Eur Arch Psychiatry Clin Neurosci. 2022 Feb 3;272(4):729–39. doi: 10.1007/s00406-021-01348-7 (PMC9095550; doi:10.1007/s00406-021-01348-7)
Supplement: Supplementary file 4 — Supplementary file4 (PDF 456 KB) [file 406_2021_1348_MOESM4_ESM.pdf]

# REPORT OF THE COMMITTEE ON METHODS OF CLINICAL EXAMINATION IN ELECTROENCEPHALOGRAPHY

1957

Before the 1953 EEG congress in Boston a committee was set up by the Council of the Federation "to review standard examination procedures in a number of the leading laboratories throughout the world, and to collect opinions with the object of formulating a generally accepted guide to EEG technique which would form a basic standard for training, and for work, in the various laboratories of the world". The report of this committee was not published because its members felt that insufficient time had been available to arrive at satisfactory conclusions.

In accordance with the statutes of the Federation its President therefore nominated a Convener to call together a new Committee. The following, several of whom had been members of the 1953 committee, accepted the invitation to serve:

W. Cobb, London, Gt. Britain. (Chairman)
C. Ajmone-Marsan, Bethesda, Maryland, U.S.A.
H. Gastaut, Marseille, France.
R. Hess, Jr., Zurich, Switzerland.
R. Jung, Freiburg, Germany.
J. R. Knott, Iowa City, U.S.A.
O. Magnus, Wassenaar, Netherlands. (Secretary)
H. Petsche, Vienna, Austria.
R. Schwab, Boston, Mass., U.S.A.
H. Terzian, Padova, Italy.
L. Widen, Stockholm, Sweden.

This Committee met several times during the period from July 17th-20th 1957, in "De Pietersberg", Oosterbeek, The Netherlands, and agreed on the Recommendations included in this report. They are based in part on the unpublished report of the previous Committee, which contained data collected by questionnaire from a great number of laboratories all over the world.

It is far from the intention of the Committee to attempt to impose a set of Rules, and the term Recommendations is chosen advisedly; at the same time, and unavoidably, the sense of these Recommendations varies considerably; some express no more than an arbitrary preference for one of a number of alternatives of possibly equal merit, others are little more than a summary of current practise, while still others outline essential principles of good recording technique. Behind all of them, however, lies the idea of facilitating the interchange of information, either by means of the original record or an illustration. If these Recommendations are followed a more or less *Standard Record* will result which will be as easily understood elsewhere as in the laboratory of its origin. This in no way restricts the right, or diminishes the need, to use additional, non-standard, techniques, nor does adherence to these Recommendations in any way reduce the need for proper training.

## RECOMMENDATIONS

A. *General*

1. A large central laboratory which serves all the departments of a hospital or several hospitals is preferable to a number of smaller ones. This enables the use of first class equipment, is more economic, provides diversified experience and training facilities, and allows the employment of one or more qualified clinical neuro-physiologists. Such a laboratory should operate as a unit under the direction of an independent clinical neuro-physiologist. Interpretative reports should not be made by technicians. The pressure of numbers of examinations should never be allowed to detract from an adequate standard of recording.

2. Adequate clinical information, including the reasons for referral, should always be provided by the referring physician. When inadequate, supplementary data should be obtained by a competent person in the laboratory before the examination. This is especially important in epileptic cases; details of the pattern of the seizure are essential for planning the examination technique to be used and the clinical interpretation of the record. Access to the clinical notes is desirable. Equally, clinical observations made during the examination should be reported to the referring physician.

3. The whole record should be stored for as long as possible, at least for 5 years.

B. *Preparation of the patient*

4. Medication should not be stopped as a routine, but only after consideration of the particular case.

5. De-greasing is generally the only necessary preparation of the scalp.

C. *Apparatus*

6. *Number of channels.* Eight channels is considered to be the minimum and sufficient number for routine clinical purposes. More channels are desirable for polygraphic recording and detailed studies.

7. *Paper speed.* Basic paper speeds should be 3 and 1.5 cm/sec. Paper speed should be varied by multiple and submultiple steps as signal characteristics demand. For special purposes a very slow paper speed may be desirable.

8. *Sensitivity* (*amplification*). The basic amplification should be 10 $\mu$V/mm. Changes should be made in steps of $\sqrt{2}$ as signal characteristics require. Every record should contain a calibration at the standard gain at the beginning and also calibrations at all gains, time constants and filters which have been used. Amplification should be indicated in $\mu$V/mm. at the beginning of each montage and at each change of gain.

9. *Time constant.* At least a part of each montage should be recorded with a time constant of not less than 0.3 sec.

10. *High frequency response.* So far as possible a part of each montage should be recorded at the full high frequency response of the apparatus.

11. Inter-electrode resistance should be checked.

12. A routine common EEG input check is desirable.

D. *Electrodes*

13. Silver pad electrodes held by a cap are quickest to apply and easiest to adjust. Electrodes fixed with collodion are less easily displaced and are probably to be preferred for restless patients, children under the age of 5 years, and examinations of long duration. For fixation of the stick-on electrodes collodion is considered superior to bentonite. Electrodes should be regularly checked for noise.

E. *Recording*

14. The 10-20 electrode system (see Appendix) is preferred. Nineteen-21 electrodes should be used for a first examination in all patients over the age of 5 years; such a number allows satisfactory separation of rhythms while not unduly reducing their amplitude. The risk of missing a local spike is considered to be minimal. Additional electrodes may be needed for precise localization.

15. Both "bipolar" and "unipolar" recording have advantages and should be used in a routine examination. Bipolar recording should always include montages with linked serial pairs in straight antero-posterior and transverse lines, preferably with large as well as with small inter-electrode distances. The dangers of misinterpretation inherent in the use of reference electrodes should be constantly kept in mind.

16. On the record, traces from the Right side should take order preference over those from the Left and a transverse sequence should read from Right to Left. In antero-posterior recordings the sequence: Right, Right, Right, Right-Left, Left, Left, Left is preferred to alternation.

17. A minimum period of 20 minutes is recommended for a first examination. A large number of channels does not permit significant reduction of this time.

18. All changes in recording parameters and the state of the patient, as well as stimulations applied, should be indicated on the record clearly and in understandable symbols (e.g. at each change of gain the resulting sensitivity should be recorded in $\mu$V/mm.).

19. The convention that a negative change of potential at grid I results in an upward pen deflection may be extended by speaking of the lead to this grid as "Black" and indicating it in diagrams by a solid line. Conversely, the lead to grid II, positivity of which causes an upward deflection, is spoken of as "White" and drawn as a broken line.

F. *Activation*

20. Each routine examination should include eye opening and closing and a period of hyperventilation of at least 3 minutes. Photic stimulation is a useful method which can easily be employed routinely. Electronic stroboscopes are without danger to the retina, which is not true of some other sources.

21. Sleep, either spontaneous or induced, is of value, particularly in convulsive disorders in children.

22. Convulsant drugs (Metrazol, Megimide, etc.) are used particularly for localization in cases of epilepsy.

Evidently this is not an exclusive list. Several methods of activation may be used advantageously in combination. It is to be noted that considerable disagreement still exists as to the specific values of the various activating procedures. In general the safest and simplest should be used first.

# APPENDIX

## THE TEN TWENTY ELECTRODE SYSTEM OF THE INTERNATIONAL FEDERATION

At the First International Congress in London in 1947 it was recommended that an attempt be made to standardize the placement of electrodes on the head for EEG examinations to facilitate comparison of records taken in different laboratories and to make it possible to have more satisfactory communication of results in the literature. Dr. Jasper was appointed to study this problem and report recommendations to the Second International Congress in Paris in 1949.

Systems of electrode placements then in current use were studied, particularly from The National Hospital, Queen Square, the system used by Dr. F. Gibbs and his colleagues in Boston and Chicago, the system developed by Drs. Schwab and Abbott at the Massachusett General Hospital in Boston, and the system then in current use at the Montreal Neurological Institute. It was found that only minor differences existed between these several systems of electrode placement, though the designations used (numbers, letters, etc.) were entirely different. It was felt, therefore, that it should be possible to design a compromise system, making use of the advantages of the various systems then in use, so that common agreement could be reached and an international standard formulated.

Certain principles were laid down as follows:

1. Positions of electrodes should be determined by measurement from standard landmarks on the skull. Measurements should be proportional to skull size and shape, insofar as possible.

2. Adequate coverage of all parts of the head should be provided with standard designated positions even though all would not be used in a given examination.

3. Designations of positions should be in terms of brain areas (Frontal, Parietal, etc.) rather than only in numbers so that communication would become more meaningful to the non-specialist.

4. Anatomical studies should be carried out to determine the cortical areas most likely to be found beneath each of the standard electrode positions in the average subject.

*Method of Measurement.*

The anterior-posterior measurements are based upon the distance between the nasion and the inion over the vertex in the mid-line. Five points are then marked along this line, designated Frontal pole (Fp), Frontal (F), Central (C), Parietal (P), and Occi-

pital (O). The first point (Fp) is 10 per cent of the nasion-inion distance above the nasion; the second point (F) is 20 per cent of this distance back from the point Fp, and so on in 20 per cent steps back for the Central, Parietal, and Occipital mid-line points (hence the name 10-20 system). These divisions are

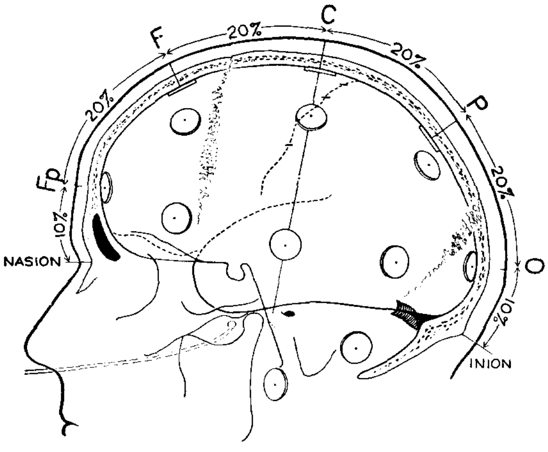


Fig. 1

Lateral view of skull to show methods of measurement from nasion to inion at the mid-line. Fp is frontal pole position, F is the frontal line of electrodes, C is the central line of electrodes, P is the parietal line of electrodes and O is the occipital line. Percentages indicated represent proportions of the measured distance from the nasion to the inion. Note that the central line is 50% of this distance. The frontal pole and occipital electrodes are 10% from the nasion and inion respectively. Twice this distance, or 20%, separates the other line of electrodes.

illustrated in figure 1. It will be noted that this places the Central line of electrodes just one half the distance between nasion and inion.

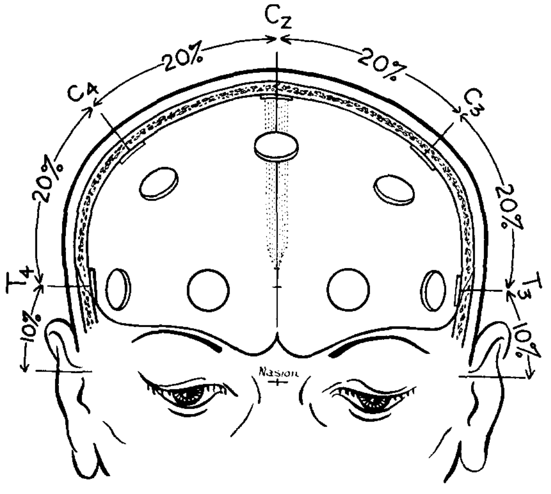


Fig. 2

Frontal view of the skull showing the method of measurement for the central line of electrodes as described in the text.

For example, if the nasion-inion distance is 30 cm. for a given patient, the Fp line will be 3 cm. above the nasion, the F line 6 cm. back of the Fp line (or 9 cm. from the nasion), the C line 6 cm. back of the F line (or 15 cm. from the nasion). There will also be 6 cm. between the C and P lines, and 6 cm. between P and O. The occipital points are then 3 cm. above the inion.

Lateral measurements are based upon the Central coronal plane. The distance is first measured from left to right preauricular points (felt as depressions at the root of the zygoma just anterior to the tragus). These points were selected because they seemed easier to determine with accuracy than the external auditory meati. Be sure the tape is passing through the predetermined Central point at the vertex when making this measurement. Ten per cent of this distance is then taken for the temporal point up from the preauricular point on either side. The Central points are then marked 20 per cent of the distance above the temporal points, as shown in figure 2.

Then A-P line of electrodes over the temporal lobe, frontal to occipital, is determined by measuring the distance between the Fp mid line point (as determined above), through the T position of the Central line, and back to the mid occipital point. The Fp electrode position is then marked 10 per cent of this distance from the mid-line in front, and the occipital electrode position 10 per cent of the distance from the mid-line in back. The inferior Frontal and posterior temporal positions then fall 20 per cent of the distance from the Fp and O electrodes respectively along this line, as shown in figure 3.

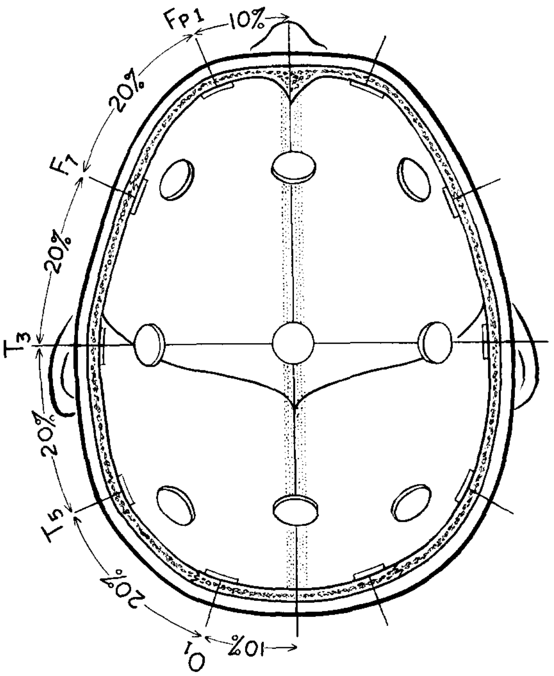


Fig. 3

Superior view with cross section of skull through the temporal line of electrodes illustrating the 10/20 system applied in this direction as described in the text.

The remaining mid-Frontal (F3 and F4) and mid-Parietal (P3 and P4) electrodes are then placed along the Frontal and Parietal coronal lines respectively, equidistant between the mid-line and temporal line of electrodes on either side, as shown in figure 4.

This provides a total of 21 standard electrode positions, including midline electrodes in Frontal, Central and Parietal regions, and the two auricular electrodes. Electrode separations are approximately the same for all pairs in the A-P direction. Coronal lines of electrodes are also approximately equally spaced, with the exception of the shorter distance between the auricular and mid-temporal points. Additional electrodes may be placed between any of these principal standard positions for especially refined localization studies (with numbers provided for these special positions as well, as indicated below).

*Designations of Electrode Positions.*

Traditional anatomical terms have been employed to designate electrode positions over the various lobes of the brain, with the exception of the Central region which is, strictly speaking, partly frontal and partly parietal. It represents the cortex in the vicinity of the Central Sulcus, both pre and post-central. It is sometimes called the *sensori-motor area.*

In order to differentiate between homologous positions over left and right hemispheres it was decided to use even numbers as subscripts for the right hemisphere, and odd numbers for the left hemisphere. Fp2, F4, F8, C4, P4, T4, T6, and O2 become standard positions on the lateral aspect of the right hemisphere, while Fpl, F3, F7, C3, P3, T3, T5 and O1 become standard lateral positions over the left hemisphere. These numbers were selected to allow for intermediate positions (e.g. F2, C2, C6, etc.) for special localization studies.

Electrodes at the mid-line in Frontal, Central and Parietal regions were originally designated Fo, Co and Po but this led to some confusion since Po, for example, might be interpreted as parieto-occipital. Consequently the midline positions have been changed to Fz, Cz, and Pz (z for zero !). The complete system of placements with designations is shown in figure 4, 5, and 6.

In addition to the positions described above pharyngeal electrodes are designated Pgl or Pg2 for the left and right side respectively. Additional electrode positions over the posterior fossae are also shown, designated Cbl and Cb2 (Cerebellar) for the left and right sides respectively.

*Anatomical Studies.*

After these electrode positions were agreed upon, anatomical studies were carried out with the help of Dr. Penfield, Dr. McRae and Dr. Caveness to determine the cortical areas over which each position would lie in the average brain. Two methods were employed: (1) metal clips placed along the Central and Sylvian fissures at operation were then used to identify these fissures in X-ray studies of the skull after the EEG electrodes had been applied, and (2) electrode positions were carefully marked in the head of cadavers, drill holes placed through the skull and the cortex marked with India ink in each position before removing the brain for examination. Brains with gross lesions or local atrophy were excluded.

Although some variability was found, and is to be expected, the position of the two principle fissures

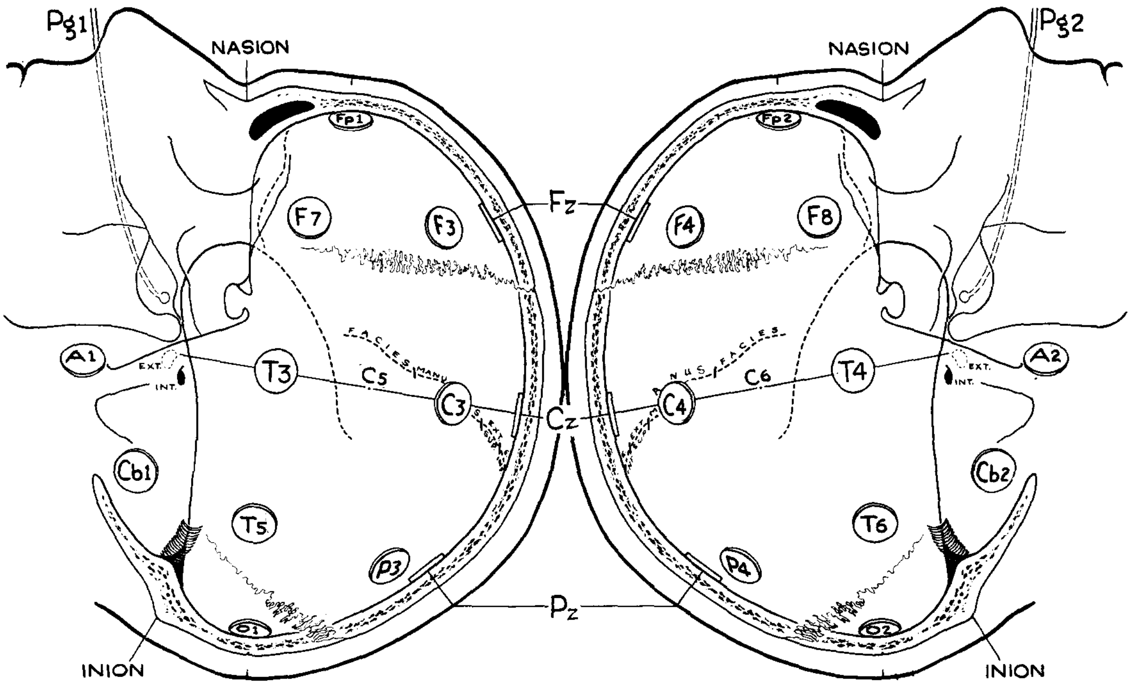


Fig. 4

The lateral view of left and right hemispheres showing all standard electrode positions, omitting intermediate positions (such as C5 and C6) which are used only for special studies with more closely spaced electrodes. These drawings were made from a series of X-ray projections with true lateral views. The location of principal fissures was determined by silver clips placed at operation and by other anatomical studies described in the text. The location of pharyngeal electrodes (Pg 1 and Pg 2) was also obtained from X-ray studies with these electrodes in place.

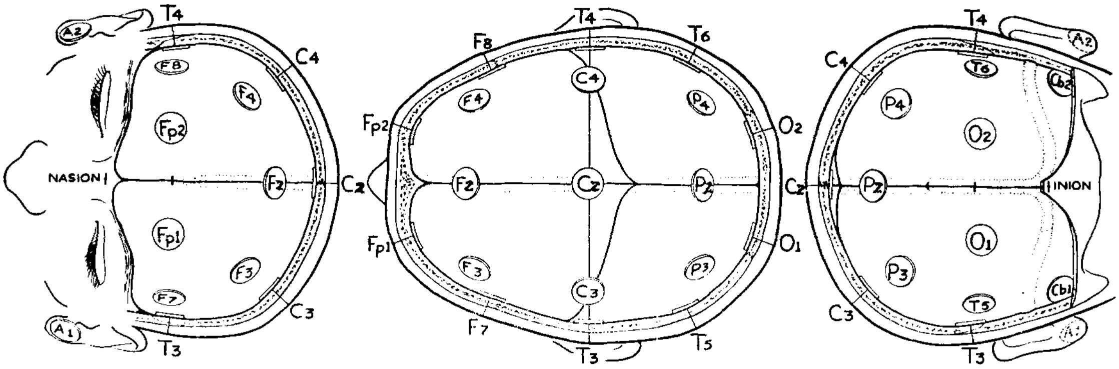


Fig. 5
Frontal superior and posterior views showing all the standard electrode positions as described in the text.

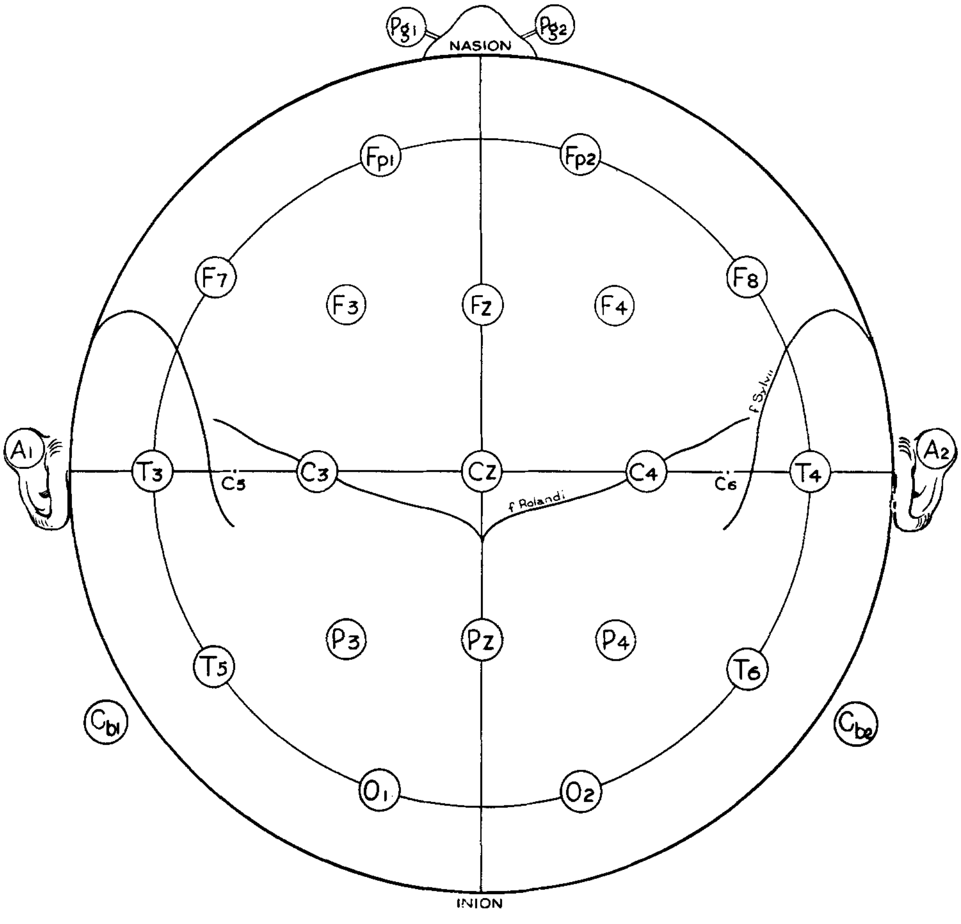


Fig. 6
A single plane projection of the head, showing all standard positions and the location of the Rolandic and Sylvian fissures. The outer circle was drawn at the level of the nasion and inion. The inner circle represents the temporal line of electrodes. This diagram provides a useful stamp for the indication of electrode placements in routine recording.

should be within plus or minus about 1 cm. of that indicated on the drawings, provided the head measurements are carefully made and the brain is free of gross distortion due to expanding or contracting lesions. Due to the obliquity of the Central Fissure the upper central electrodes will usually lie pre-central while the lower ones will be post-central in most cases.

## COMMENTS

This electrode system was adopted for trial at the meeting of the General Assembly of the International Federation in Paris, 1949. Since then it has been adopted by several laboratories and has been considered fairly satisfactory. It was not intended that this system should prevent trials of other electrode placements, possibly with the view of its revision at a subsequent International Congress. It should help to make more comparable the results obtained in various laboratories. It should certainly facilitate the communication between laboratories, in the literature, and with referring physicians who become familiar with the localization of EEG abnormalities in terms of these standard landmarks.

It should be pointed out that this is not "the Montreal system" or "the Jasper system" as it has sometimes been erroneously called. A different method of measurement was used in the Montreal Laboratories prior to 1949 when it was changed to conform to the International Standard recommendation.

Respectfully submitted,

Herbert H. Jasper, M.D.

PRINTED IN CANADA
