# Supplementary material for: The effects of hypnotherapy compared to cognitive behavioral therapy in depression: a NIRS-study using an emotional gait paradigm
Source: Eur Arch Psychiatry Clin Neurosci. 2022 Feb 3;272(4):729–39. doi: 10.1007/s00406-021-01348-7 (PMC9095550; doi:10.1007/s00406-021-01348-7)
Supplement: Supplementary file 5 — Supplementary file5 (PDF 286 KB) [file 406_2021_1348_MOESM5_ESM.pdf]

ACADEMIA

Accelerating the world's research.

How Psychotherapy Changes the Brain Understanding the Mechanisms

Luciano Restrepo

Related papers

Download a PDF Pack of the best related papers

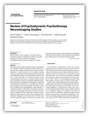

Review of Psychodynamic Psychotherapy Neuroimaging Studies
Sarah Nowoweiski

Evidence Why Paroxetine Dose Escalation is Not Effective in Major Depressive Disorder: A Randomize…
Henricus Ruhe

Effect of the selective serotonin reuptake inhibitor paroxetine on platelet function is modified by a SL…
David Adson

Psychiatric Times

Psychiatric Times. Vol. 28 No. 8
PSYCHOTHERAPY UPDATE

How Psychotherapy Changes the Brain

Understanding the Mechanisms

By Hasse Karlsson, MA, MD, PhD | August 11, 2011
Dr Karlsson is Professor of Psychiatry at the University of Helsinki and Professor of Integrative Neuroscience and Psychosomatics at the University of Turku in Finland. He reports that he has received honoraria from Lundbeck and Janssen-Cilag; he is a board member of Psychiatric Research and Treatment Station EOS.

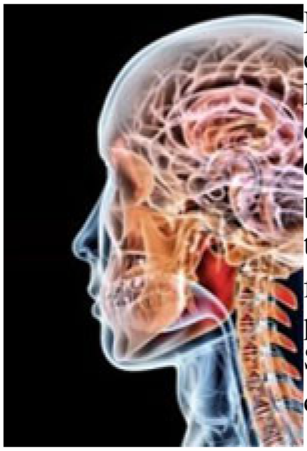

Psychotherapy outcomes and the mechanisms of change that are related to its effects have traditionally been investigated on the psychological and social levels, by measuring changes in symptoms, psychological abilities, personality, or social functioning. Many psychiatrists have also held the unfortunate dichotomized position that psychotherapy is a treatment for “psychologically based” disorders, while medication is for “biologically based” disorders.[1] During the past several decades, it has become clear that all mental processes derive from mechanisms of the brain.[2] This means that any change in our psychological processes is reflected by changes in the functions or structures of the brain. Straightforward reductionistic stances, however, are unfounded because there is clear evidence that our subjective experiences affect the brain.[1]

Plastic changes in the brain have been difficult to study in humans, but there has been more success in animal studies. Changes in the brain in relation to experience have been detected at the cellular and molecular levels in animals using different experimental approaches. The advent of functional neuroimaging, including single photon emission CT (SPECT), positron emission tomography (PET), and functional MRI, has made it possible to study changes at the brain systems level (by measuring changes in brain blood flow or metabolisms) and, increasingly, also on the molecular level using SPECT and PET in the living human brain.

Brain system level studies on the effects of psychotherapy

So far, nearly 20 studies on brain changes after psychotherapy for depression, anxiety disorders, and borderline personality disorder have been published (**Table**). The first study was published nearly 20 years ago, in 1992. In this study, the researchers compared behavior therapy with fluoxetine(Drug information on fluoxetine) treatment. Both treatment modalities demonstrated similar changes in the brain—especially in the caudate nucleus.

Drawn together, these system level studies suggest that cognitive-behavioral therapy (CBT), dialectic behavior therapy (DBT), psychodynamic psychotherapy, and interpersonal psychotherapy alter brain function in patients suffering from major depressive disorder (MDD), obsessive-compulsive disorder, panic disorder, social anxiety disorder, specific phobias, posttraumatic stress disorder, and borderline personality disorder (BPD).[3-16]

Table: Studies on brain changes after psychotherapy for depression, anxiety disorders, and borderline personality disorder

Study	Diagnosis	No. of patients	Imaging method	Treatment
Baxter[6]	OCD	18	FDG-PET	BT vs fluoxetine
Schwartz[8]	OCD	9	FDG-PET	CBT
Martin[4]	MDD	28	HMPAO-SPECT	IPT vs venlafaxine
Brody[3]	MDD	24	FDG-PET	IPT vs paroxetine
Furmark[11]	Social phobia	18	^{15}O-PET	CBT vs citalopram
Nakatani[7]	OCD	31	Xe-CT	BT
Paquette[12]	Spider phobia	12	fMRI	CBT
Goldapple[5]	MDD	17	FDG-PET	CBT
Praško[9]	PD	12	FDG-PET	CBT vs antidepressants
Nakao[29]	OCD	10	fMRI	CBT vs fluvoxamine
Sakai[30]	PD	12	FDG-PET	CBT
Straube[13]	Spider phobia	28	fMRI	DBT
Schnell[15]	BPD	6	fMRI	DBT
Lai[16]	BPD	2	SPECT	PDT
Felmingham[14]	PTSD	8	fMRI	CBT
Lehto[20]	MDD	19	SPECT	PDT vs waiting list
Beutel[10]	PD	9	fMRI	PDT
Apostolova[31]	OCD	16	FDG-PET	CBT vs paroxetine
Karlsson[17]	MDD	9	WAY-PET	Short-term PDT vs fluoxetine

OCD, obsessive-compulsive disorder; FDG-PET, [18F]-fluorodeoxyglucose positron emission tomography; BT, behavior therapy; CBT, cognitive-behavioral therapy; MDD, major depressive disorder; HMPAO-SPECT, ^{99m}Tc–hexamethylpropyleneamine oxime single photon emission CT; IPT, interpersonal psychotherapy; ^{15}O-PET, oxygen-15 positron emission tomography; Xe-CT, xenon-enhanced CT; BT, behavioral therapy; fMRI, functional MRI; PD, panic disorder; DBT, dialectic behavior therapy; BPD, boderline personality disorder; SPECT, single photon emission CT; PDT, psychodynamic psychotherapy; PTSD, posttraumatic stress disorder; WAY-PET, positron emission tomography using [carbonyl-11C] WAY-100635.

The majority of these studies have reported similar brain changes after psychotherapy and medication. However, some recent studies have also shown clear differences among these treatment modalities. In the study by Goldapple and colleagues,[5] treatment response for CBT in patients with MDD was associated with increases in metabolism in the hippocampus and dorsal cingulate and decreases in the dorsal, ventral, and medial frontal cortex. This pattern was clearly distinct from the pattern caused by paroxetine(Drug information on paroxetine), which included increases in metabolism in the prefrontal areas and decreases in the hippocampus and subgenual cingulate. In the recent study by Karlsson and colleagues,[17] clear differences emerged between short-term psychodynamic psychotherapy and fluoxetine among patients with MDD.

From brain changes to mechanisms of psychotherapy

In addition to just reporting the findings on brain changes as a result of psychotherapy, some of these studies have made it possible to construct models that explain the mechanisms behind the changes that result from the different psychotherapies. These models can then be compared with the psychological theories of these psychotherapies.

Many psychotherapies attempt to enhance patients' problem-solving capacities, self-representation, and regulation of affective states. The brain areas that play a role in these functions include the dorsolateral prefrontal cortex, ventral anterior cingulate cortex, dorsal anterior cingulate cortex, ventral and dorsal subregions of the medial prefrontal cortex, posterior cingulate cortex, precuneus, insular cortex, amygdala, and ventrolateral prefrontal cortex. (For a review, see Frewen et al.[18])

For example, the mechanism behind the effectiveness of cognitive therapy for patients with MDD could be through an increase in prefrontal function, which is involved in cognitive control, while antidepressant medications operate more directly on the amygdala, which is involved in the generation of negative emotion.[19]

Because one of the major hypotheses regarding the effect of CBT on brain functioning concerns a more effective ("top-down") regulation of hyperexcitable limbic structures by prefrontal control systems, it seems that psychodynamic psychotherapy may also function, at least in part, via these mechanisms. The findings from a study by Beutel and colleagues[10] are in line with this hypothesis and demonstrate both a frontal deactivation and an amygdala-hippocampal hyperactivation seen in symptomatic patients with panic disorder. When their panic symptoms and anxiety levels were reduced following treatment, the frontal deactivation and amygdala-hippocampus hyperactivation were normalized.

Affective hyperarousal is the hallmark of BPD and the main target for DBT. This would mean that DBT leads to a decrease in the activity in relation to emotional stimuli in the brain areas that serve these

functions. This was indeed found in the study by Schnell and Herpertz,[15] whose findings suggest that after DBT, there is a decrease in hemodynamic response to negative stimuli in the right-sided anterior cingulate, the temporal and posterior cingulate cortices, and the left insula.

Molecular psychodynamics

All these studies, however, have investigated the brain changes on the whole brain systems level. To understand the more basic mechanisms related to psychotherapy, possible molecular and cellular changes should also be studied. So far, only 2 Finnish studies have measured molecular level changes after psychotherapy, and in this way directly tested the hypothesis put forth by Kandel[2] that psychotherapy could lead to changes in gene expression through learning, by altering the strength of synaptic connections between nerve cells and inducing morphological changes in neurons. Interestingly, in both studies, the psychotherapy that was used was psychodynamic.

In the study by Lehto and colleagues,[20] 19 depressive outpatients received psychodynamic psychotherapy for 12 months. Of the patients, 8 were classified as having atypical depression. Midbrain serotonin transporter and striatum dopamine(Drug information on dopamine) transporter densities were recorded using SPECT brain imaging with the [123I]nor--CIT radioligand before and after psychotherapy. The researchers showed that midbrain serotonin transporter density significantly increased during psychotherapy in patients with atypical depression, but not among patients with standard depression. There were no changes in the levels of striatum dopamine transporter. Because of the subgroup finding, these results are difficult to interpret, and one of the shortcomings of this study is the lack of a control group.

Figure — Visualization of the results from the SPM analysis

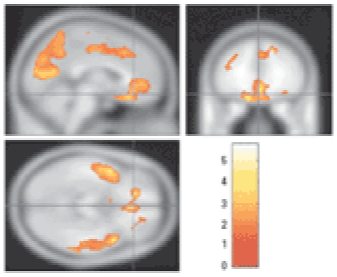

The analysis of parametric [carbonyl-11C]WAY-100635 binding potential (BP_{ND}) maps at the voxel level showed 2 large clusters [k_E = 9515, T_{max} = 5.82 at (−26, 0, −38) and k_E = 18,231 voxels, T_{max} = 4.10 at (−46, −20, 42)] located mainly in frontal, temporal, and parietal cortices. These clusters represent significantly increased BP_{ND} in the psychotherapy group compared with the fluoxetine group. The results are visualized on a T1-weighted MRI template in stereotactic standard space; the color bar represents the T statistic at voxel level.

SPM, statistical parametric mapping.
Reproduced with permission from Nature Reviews Neuroscience, 2008, vol 9.

In the other Finnish study, patients with MDD were randomized to receive either short-term psychodynamic psychotherapy or fluoxetine. Before being treated and after 4 months of treatment, they underwent a brain scan with PET using [carbonyl-11C]WAY-100635 (measures the density of serotonin type 1A [5-HT_{1A}] receptors) and [11C]raclopride (measures density of dopamine type 2/3 receptors). In the 2 published papers, the researchers reported that the clinical outcome in both treatment groups was similar in terms of standard symptom ratings (symptom remission was achieved in 59% of the patients and 77% of the patients met criteria for response).[17,21] However, an analysis of the change in the 5-HT_{1A} receptor density in the treatment groups revealed a significant increase in the psychotherapy group compared with the medication group, for which no change was detected (**Figure**). Fluoxetine increased [11C]raclopride binding in the lateral thalamus; no change was seen in the group that received psychotherapy.

Several previous studies have found changes in 5-HT_{1A} receptor binding in MDD that is not reversed by SSRI treatment.[22-27] This could mean that the recovery process in MDD after psychotherapy is different from recovery after medication. Currently, the clinical implications of these findings are unknown, but they may be related to the finding that suggests that the relapse rate for MDD is lower in patients treated with psychotherapy than in those treated with antidepressants.[28]

Conclusion

Although still preliminary, the studies using neuroimaging for measuring change caused by psychotherapy will in the long run lead to a more refined understanding of how different psychotherapies work. This may lead to a development in which specific modes of psychotherapy can be designed to target specific brain circuits.[1] In addition, neurobiological research may help refine psychological theories about the change processes.

References

1. Gabbard GO. A neurobiologically informed perspective on psychotherapy. *Br J Psychiatry*. 2000;177:117-122.
2. Kandel ER. A new intellectual framework for psychiatry. *Am J Psychiatry*. 1998;155:457-469.
3. Brody AL, Saxena S, Stoessel P, et al. Regional brain metabolic changes in patients with major depression treated with either paroxetine or interpersonal therapy: preliminary findings. *Arch Gen Psychiatry*. 2001;58:631-640.
4. Martin SD, Martin E, Rai SS, et al. Brain blood flow changes in depressed patients treated with interpersonal psychotherapy or venlafaxine hydrochoride: preliminary findings. *Arch Gen Psychiatry*. 2001;58:641-648.
5. Goldapple K, Segal Z, Garson C, et al. Modulation of cortical-limbic pathways in major depression: treatment-specific effects of cognitive behavior therapy. *Arch Gen Psychiatry*. 2004;61:34-41.
6. Baxter LR Jr, Schwartz JM, Bergman KS, et al. Caudate glucose metabolic rate changes with both drug and behavior therapy for obsessive-compulsive disorder. *Arch Gen Psychiatry*. 1992;49:681-689.
7. Nakatani E, Nakgawa A, Ohara Y, et al. Effects of behavior therapy on regional cerebral blood flow in obsessive-compulsive disorder. *Psychiatry Res*. 2003;124:113-120.
8. Schwartz JM, Stoessel PW, Baxter LR, et al. Systematic changes in cerebral glucose metabolic rate after successful behavior modification treatment of obsessivecompulsive disorder. *Arch Gen Psychiatry*. 1996;53:109-113.
9. Praako J, Horácek J, Zálesky R, et al. The change of regional brain metabolism (18FDG PET) in panic disorder during the treatment with cognitive behavioral therapy or antidepressants. *Neuro Endocrinol Lett*. 2004;25:340-348.
10. Beutel ME, Stark R, Pan H, et al. Changes of brain activation pre- post short-term psychodynamic inpatient psychotherapy: an fMRI study of panic disorder patients. *Psychiatry Res*. 2010;184:96-104.
11. Furmark T, Tillfors M, Marteinsdottir I, et al. Common changes in cerebral blood flow in patients with social phobia treated with citalopram or cognitive-behavioral therapy. *Arch Gen Psychiatry*. 2002;59:425-433.
12. Paquette V, Lévesque J, Mensour B, et al. Change the mind and you change the brain: effects of cognitive-behavioral therapy on the neural correlates of spider phobia. *Neuroimage*. 2003;18:401-409.
13. Straube T, Glauer M, Dilger S, et al. Effects of cognitive-behavioral therapy on brain activation in specific phobia. *Neuroimage*. 2006;29:125-135.
14. Felmingham K, Kemp A, Williams L, et al. Changes in anterior cingulate and amygdala after cognitive behavior therapy of posttraumatic stress. *Psychol Sci*. 2007;18:127-129.
15. Schnell K, Herpertz SC. Effects of dialectic-behavioral-therapy on the neural correlates of affective hyperarousal in borderline personality disorder. *J Psychiatr Res*. 2007;41:837-847.
16. Lai C, Daini S, Calcagni ML, et al. Neural correlates of psychodynamic psychotherapy in borderline disordersa pilot investigation. *Psychother Psychosom*. 2007;76:403-405.

17. Karlsson H, Hirvonen J, Kajander J, et al. Research letter: psychotherapy increases brain serotonin 5-HT1A receptors in patients with major depressive disorder. *Psychol Med*. 2010;40:523-528.
18. Frewen PA, Dozois DJ, Lanius RA. Neuroimaging studies of psychological interventions for mood and anxiety disorders: empirical and methodological review. *Clin Psychol Rev*. 2008;28:228-246.
19. DeRubeis RJ, Siegle GJ, Hollon SD. Cognitive therapy versus medication for depression: treatment outcomes and neural mechanisms. *Nat Rev Neurosci*. 2008;9:788-796.
20. Lehto SM, Tolmunen T, Joensuu M, et al. Changes in midbrain serotonin transporter availability in atypically depressed subjects after one year of psychotherapy. *Prog Neuropsychopharmacol Biol Psychiatry*. 2008;32:229-237.
21. Hirvonen J, Hietala J, Kajander J, et al. Effects of antidepressant drug treatment and psychotherapy on striatal and thalamic dopamine D2/3 receptors in major depressive disorder studied with [11C]raclopride PET. *J Psychopharmacol.* 2010 Sep 9; [Epub ahead of print].
22. Drevets WC, Frank E, Price JC, et al. PET imaging of serotonin 1A receptor binding in depression. *Biol Psychiatry*. 1999;46:1375-1387.
23. Sargent PA, Kjaer KH, Bench CJ, et al. Brain serotonin 1A receptor binding measured by positron emission tomography with [11C]WAY-100635: effects of depression and antidepressant treatment. *Arch Gen Psychiatry*. 2000;57:174-180.
24. Bhagwagar Z, Wylezinska M, Taylor M, et al. Increased brain GABA concentrations following acute administration of a selective serotonin reuptake inhibitor. *Am J Psychiatry*. 2004;161:368-370.
25. Meltzer CC, Price JC, Mathis CA, et al. Serotonin 1A receptor binding and treatment response in late-life depression. *Neuropsychopharmacology*. 2004;29:2258-2265.
26. Parsey RV, Oquendo MA, Ogden RT, et al. Altered serotonin 1A binding in major depression: a [carbonyl-C-11]WAY100635 positron emission tomography study. *Biol Psychiatry*. 2006;59:106-113.
27. Moses-Kolko EL, Wisner KL, Price JC, et al. Serotonin 1A receptor reductions in postpartum depression: a positron emission tomography study. *Fertil Steril*. 2008;89:685-692.
28. Hollon SD, Jarrett RB, Nierenberg AA, et al. Psychotherapy and medication in the treatment of adult and geriatric depression: which monotherapy or combined treatment? *J Clin Psychiatry*. 2005;66:455-468.
29. Nakao T, Nakagawa A, Yoshiura T, et al. Brain activation of patients with obsessive-compulsive disorder during neuropsychological and symptom provocation tasks before and after symptom improvement: a functional magnetic resonance imaging study. *Biol Psychiatry*. 2005;57:901-910.
30. Sakai Y, Kumano H, Nishikawa M, et al. Changes in cerebral glucose utilization in patients with panic disorder treated with cognitive-behavioral therapy. *Neuroimage*. 2006;33:218-226.
31. Apostolova I, Block S, Buchert R, et al. Effects of behavioral therapy or pharmacotherapy on brain glucose metabolism in subjects with obsessive-compulsive disorders as assessed by brain FDG-PET. *Psychiatry Res*. 2010;184:105-116.
